# Beyond the Classical Electron‐Sharing and Dative Bond Picture: Case of the Spin‐Polarized Bond

**DOI:** 10.1002/anie.202010948

**Published:** 2020-11-11

**Authors:** Pedro Salvador, Eva Vos, Inés Corral, Diego M. Andrada

**Affiliations:** ^1^ Institut de Química Computacional i Catàlisi i Departament de Química Universitat de Girona c/M Aurelia Capmany 69 17003 Girona Spain; ^2^ Departamento de Química Facultad de Ciencias Módulo 13, and Institute of Advanced Chemical Sciences (IadChem) Universidad Autónoma de Madrid, Campus de Excelencia UAM-CSIC, Cantoblanco 28049 Madrid Spain; ^3^ Faculty of Natural Sciences and Technology Department of Chemistry Saarland University 66123 Saarbrücken Germany

**Keywords:** bond analysis, dative bonds, donor-acceptor systems, electronic structure, sodium

## Abstract

Chemical bonds are traditionally assigned as electron‐sharing or donor‐acceptor/dative. External criteria such as the nature of the dissociation process, energy partitioning schemes, or quantum chemical topology are invoked to assess the bonding situation. However, for systems with marked multi‐reference character, this binary categorization might not be precise enough to render the bonding properties. A third scenario can be foreseen: spin polarized bonds. To illustrate this, the case of a NaBH_3_^−^ cluster is presented. According to the analysis NaBH_3_^−^ exhibits a strong diradical character and cannot be classified as either electron‐sharing or a dative bond. Elaborated upon are the common problems of popular bonding descriptions. Additionally, a simple model, based on the bond order and local spin indicators, which discriminates between all three bonding situations, is provided.

## Graphical abstract

Conventional chemical bonding analysis distinguishes between two situations: electron sharing and donor‐acceptor. However, a third scenario is possible where the bond suffers spin polarization. Discussed here is the case of NaBH_3_^−^, better classified as a spin‐polarized bond. A simple model to distinguish between these bonding types is provided.

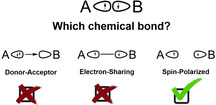

The chemical bond is a central paradigm for describing molecular structure and reactivity.^[1]^ A fundamental approach towards understanding its properties consists in classifying the electron‐pair interactions between atoms or fragments.^[2]^ There are two well‐established classes of bonding interactions, according to the origin of the electron‐pair. When each fragment contributes with one electron, the bonding is described as an electron‐sharing bond. When both electrons are contributed by one of the fragments, the interaction is interpreted as a dative or donor‐acceptor bond.^[3]^

The IUPAC recommends to analyse the nature of the chemical bond considering the minimum‐energy rupture in the gas phase or in inert solvents.^[4]^ Following Haaland's guidelines, a bond is classified as dative if the minimum energy bond rupture proceeds heterolytically, while it is an electron‐sharing bond if this rupture proceeds homolytically.^[5]^ Such a distinction oversees the electronic rearrangement happening during dissociation. Therefore, some systems can lead to heterolytic dissociation despite the fact that each fragment contributes with one electron.^[6]^ Several methods based on valence bond theory, topological analysis, and molecular orbital theory have been used to assess the bonding interaction “without” the need of dissociation, but in most cases recurring to a seemingly unavoidable arbitrary fragmentation.^[7]^

In general, it is not trivial (maybe impossible) to distinguish between a dative and an electron‐sharing situation, without invoking an external criterion.[^7^, ^8^] Arguably, the orbital‐based method energy decomposition analysis (EDA) and the quantum chemical topology (QCT) approaches are considered successful methods to solve such a task.^[9]^ Within the EDA scheme, the bond is decomposed into an electrostatic interaction (Δ*E*_elstat_) between the frozen‐density fragments, the Pauli repulsion (Δ*E*_Pauli_) associated to the antisymmetrization of the wave function, and the stabilizing orbital term (Δ*E*_orb_), accounting for the final orbital relaxation. These terms depend on the specific reference *electronic state* of the fragments, thus there is no exclusive bond fragmentation. The lower the absolute values of the orbital term (Δ*E*_orb_), the better the representation of the chemical bond since this translates into a lower reorganization degree.^[10]^ Aside from being a path function, this method depends on the correct representation of the ground state.^[11]^ With QCT methods, specifically the atoms in molecules (QTAIM) approach, the value of different descriptors at the bond critical point are used to assess the nature of the chemical bond.^[12]^ Since no reference states are needed, this method avoids the inherent problems carried by fragmentation schemes. Although physically well‐founded, it lacks predictive power and is prone to misinterpretations when it is connected with heuristic orbital models.^[13]^

Considering the AB system interacting through a dative bond, if A: is the donor and B is the acceptor, the contributions in terms of electron population of A: and B to the A−B bond would be 2‐δ and δ, respectively, where δ accounts for the donation of electron density upon bond formation. Instead, if the bonding interaction is electron‐sharing the atomic populations of A**^.^** and B**^.^** would be (assuming *χ*_A_>*χ*_B_) *N*_A_=1+*p* and *N*_B_=1−*p*, where *p* accounts for the bond polarization, induced by the different local electronegativity (*χ*) of A and B. Both pictures are naturally related by δ + *p*=1. When *p* → 0, the electron‐sharing fragmentation would likely lead to a smaller orbital interaction than the donor‐acceptor one. The contrary is expected as *p* → 1.

However, one can envisage a third scenario, where the bond suffers from spin polarization. In that case, the α and β atomic populations would be defined as *N*_A_^α^=1+*p* ^α^, *N*_B_^α^=1−*p* ^α^, *N*_A_^β^=1−*p* ^β^ and *N*_B_^β^=1+*p* ^β^. The spin density on each fragment will be given by *p_s_*=|*p* ^α^+*p* ^β^|, while the overall bond polarization *p*=|*p* ^α^−*p* ^β^| would likely be small.

Spin polarization in bonds is a well‐documented phenomenon. For instance, high‐valent oxo‐iron species are key intermediates in the catalytic cycles of oxygen activating iron enzymes such as the cytochrome P450. The extent of spin polarization of the Fe=O unit stands behind debates over its electronic structure, namely oxo‐iron(IV) vs. oxyl‐iron(III) pictures.^[14]^ In nitrosyl chemistry, spin polarization also plays a major role when it comes to assigning the oxidation states of the metal‐NO unit.^[15]^ It also hinders the rationalization of metal‐metal multiple bonding.^[16]^

In the extreme case, spin‐polarization leads to a diradical species. Intermediate situations are usually referred as diradicaloids. Signatures of diradical character are a small singlet‐triplet gap and a spin‐polarized (broken‐symmetry, BS) solution below the closed‐shell (CS) description of single determinantal methods. In fact, incorporating static correlation is pivotal for the correct description of spin polarization.

In most EDA approaches, spin‐polarization in the fragments and the spin‐coupled intermediate state is not properly considered, with exceptions.^[17]^ Importantly, the appearance of a BS solution below the CS one, increments the Δ*E*_orb_ values for the donor‐acceptor and electron‐sharing patterns by the same amount. The other terms, namely Δ*E*_elstat_, Δ*E*_Pauli_ and Δ*E*_prep_, keep the same magnitude. If the intermediate state, built up from A: + B, is higher in energy than that from A^.^ + ^.^B, irrespective of the nature of the ground‐state of AB, the lowest Δ*E_orb_* criterion would necessarily point towards an “electron‐sharing” situation, or better said, to a reference state with one electron per fragment. Hence, such a criterion appears to be useful merely to discriminate the dative picture from the other two. EDA is not designed to distinguish a classical electron‐sharing from a spin‐polarized interaction and, in the limiting case, from a diradical!

More suitable bonding indicators are bond orders and particularly the local spin.^[18]^ In Mayer's local spin analysis (LSA), the expectation value of the spin‐squared operator is decomposed into atomic (local spins) and diatomic terms. The most relevant feature of LSA is that, even for pure singlet states, the method is able to differentiate a CS covalent molecule from an anti‐ferromagnetic system in which the local spins are coupled to a singlet, and intermediate situations. For the previously discussed A‐B interaction, in the limiting case of having a perfect singlet diradical, one would expect the local spins to be ⟨S^2^⟩_A_=⟨S^2^⟩_B_=3/4 and the diatomic term to amount to ⟨S^2^⟩_AB_=−3/4, indicating a perfect entanglement of the electrons.^[10]^

Considering a simple two‐electron single‐determinant minimal basis model for the AB system, the CS description leads to a Mayer's bond order of 1−*p* ^2^, that is, the covalent bond order decreases with the square of the bond polarization. The interaction can be considered as perfectly covalent as the local spin trivially vanishes. When spin polarization is allowed (via BS), the Mayer bond order varies as 1−*p* ^2^−*p*_s_^2^, where *p_s_* indicates the spin polarization amount, that is, both bond polarization and spin polarization are responsible for the decrease of the bond order, at the same ratio. In the absence of bond polarization, the local spin amounts to ⟨S^2^⟩_A_=3/4 *p*_s_^2^ (1−S_AB_^2^), where S_AB_ is the atomic overlap. That is, the increase of local spin is concomitant with the decrease of the covalent bond order due to spin polarization. Deviations from classical covalent bonding with increasing local spin have been observed for correlated wave functions.^[19]^ Thus, the combined consideration of both bond order and local spin indicators affords the distinction between all three aforementioned bonding situations, as sketched in Table 1.

**Table 1:** Chemical bonding analysis

Chemical Bond	Bond order A‐B	Local spin on A and B	EDA A→B vs A–B
Electron‐sharing	Large	Small/Null	|ΔEorb(A^.^ + ^.^B)|<|ΔEorb(A: + B)|
Donor‐acceptor	Small	Small/Null	|ΔEorb(A^.^ + ^.^B)|>|ΔEorb(A: + B)|
Spin‐Polarized	Small	Medium/Large	|ΔEorb(A^.^ + ^.^B)|<|ΔEorb(A: + B)|

Let us illustrate the issue with a controversial example. Liu et al.^[20]^ have reported the realization of a NaBH_3_^−^ cluster featuring a Na−B bond. By combining anion photoelectron spectroscopy and bond dissociation energies (BDE), the authors claimed the bond as *dative* Na^−^→BH_3_. Later, Pan et al.^[21]^ on the basis of EDA as sketched on Figure 1 reinterpreted the complex as a classical electron‐sharing covalent Na‐BH_3_^−^ bond. Only recently, and based on quantum chemical topological approaches, Foroutand‐Nejad classified Na‐B as an ionic enforced covalent bond, arguing that coulombic forces between the metal and the Hs direct the interaction.^[22]^

**Figure 1:**
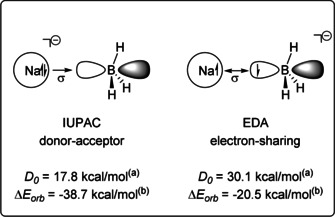
Orbital interactions (Δ*E*_orb_) and dissociation energies (*D_0_*) in NaBH_3_^−^. ^a^Ref. [20b]. ^b^Ref. [21].

As already observed by Liu et al.,^[20]^ the NaBH_3_^−^ exhibits a close‐lying triplet state of *C*_s_ symmetry. The singlet‐triplet gap obtained with the functionals used in the previous studies,[^20a^, ^21^] i.e., BP86 M06L, PBE0 are just 6.4, 1.5 and 1.7 kcal mol^−1^, respectively, in line with the CCSD(T) and CASPT2 results (5.2 and 4.3 kcal mol^−1^). Remarkably, as shown in Table 2 (Table S1), for HF, MP2 and some double‐hybrid functionals, the triplet state lies below the CS singlet state.

**Table 2:** Triplet and open shell BS electronic energies (in kcal mol^−1^) relative to the CS state and Na‐B equilibrium distance (R_e_ in Å) for NaBH_3_^−^. ⟨S^2^⟩ and diradical character (*n*_rad_).^[a]^

Method^b^	*CS (C*_3*v*_ *)*	*T (C_s_)*		*BS (C*_3*v*_ *)*			
	*R_e_*	Δ*E*_T_	*R_e_*	Δ*E*_BS_	*R_e_*	⟨S^2^⟩	*n*_rad_ [%]
HF	4.865	−7.4	2.557	−7.8	2.797	0.89	67
MP2	2.763	−3.9	2.581	−4.8	2.710	0.90	68^[c]^
CCSD(T)	2.719	5.2	2.580				0.14^[d]^
CASPT2	2.666	4.3	2.552				56^[e]^
BP86	2.707	6.4	2.579	−0.4	2.702	0.30	16
M06L	2.699	1.5	2.482	−2.9	2.668	0.71	46
M06‐2X	2.698	3.9	2.536	−3.8	2.701	0.55	33
PBE0	2.743	1.7	2.536	−2.8	2.681	0.61	37
B2PLYP	2.753	−0.4	2.562	−4.1	2.732	0.70	45

[a] Computed from ⟨S^2^⟩ as described in Ref. [25]. [b] Combined with AVTZ, except for CASSCF (AVQZ). [c] From ⟨S^2^⟩ of the HF wavefunction. [d] Largest *t*_2_ amplitude. [e] Derived from the CI coefficient of the doubly‐excited configuration.

Clearly unnoticed, the CS description of NaBH_3_^−^ *is not a stable solution*. The stability analysis^[23]^ on the CS calculations revealed the presence of an unrestricted Broke Symmetry (BS) solution that leads to a lower electronic state by 0.4 to 8.2 kcal mol^−1^, depending on the functional. Noteworthy, the BS singlet solution lies below the triplet state in all cases (Table 2). In general, the BS equilibrium distances are also in better agreement with the high‐level CCSD(T) and CASPT2 results.

The BS description should come as no surprise due to the pronounced multi‐reference character of this system.[^20^, ^22^] We have investigated the lowest singlet and triplet electronic states of NaBH_3_^−^ at the CASPT2 level. The CI coefficients for the CS and HOMO–LUMO double excited (22202000) configurations are *c*_0_=0.9009 and *c_d_*=−0.3963, and Truhlar M diagnostic^[24]^ amounts to 0.3, thus confirming the strong multi‐determinant nature of the system (Table S3). Noteworthy, the HOMO and LUMO consist of σ bonding and σ* anti‐bonding interactions between the Na 3s and BH_3_ A_1_ orbitals, which show fractional occupation numbers, as Figure 2 illustrates.

**Figure 2:**
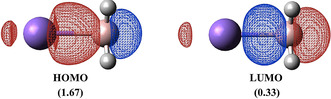
CASSCF natural orbitals and occupations at the ground state CASPT2 equilibrium structure.

The ⟨*S* ^2^⟩ values of the BS states can be used as a global indicator of diradical character (*n*_rad_).^[25]^ When a multi‐configurational wave function is used, *n*_rad_ can be derived from the weights of appropriate configurations of the CI expansion. The values summarized in Table 2 suggest non‐negligible, although largely functional dependent, *n*_rad_. While for some GGA functionals *n*_rad_ is about 16 % (BP86 or PBE), in the case of the double‐hybrid functionals *n*_rad_ reaches 50 %, in line with the wave function methods.

We have also carried out EDA for both the CS and BS descriptions at PBE0/QZ4P (Table 3 and Tables S6,S7). For both the CS and BS solutions, the orbital term for the fragmentation Na(*s* ^1^) and BH_3_^−^(A_1_^1^) is lower than for Na^−^(*s* ^2^) and BH_3_(A_1_^0^). Thus, the EDA interpretation remains unchanged, no matter the electronic state used. However, it is important to highlight that the CS results are linked to a misrepresentation of the electronic structure of the system, where two electrons are forced to occupy the σ‐bonding orbital, while the electronic structure of the BS description shows hints of deviation from a classical electron‐sharing bond.

**Table 3:** EDA of NaBH_3_^−^ for CS and BS at PBE0/QZ4P//CCSD(T)/AVTZ. Energies in kcal mol^−1^.

	CS		BS	
	Na^−^(s^2^); BH_3_(A_1_^0^)	Na(s^1^); BH_3_^−^(A_1_^1^)	Na^−^(s^2^); BH_3_(A_1_^0^)	Na(s^1^); BH_3_^−^(A_1_^1^)
Δ*E*_int_	−18.5	−29.5	−21.1	−32.1
Δ*E*_Pauli_	33.4	30.7	33.4	30.7
Δ*E*_elstat_	−17.1	−43.6	−17.1	−43.6
Δ*E*_orb_	−34.8	**−*16.6***	−37.4	**−*19.2***
Δ*E*_prep_	1.4	12.3	1.4	12.3
*D_e_*	17.1	17.1	19.8	19.8

In the above minimal basis AB model, the three bonding scenarios translate into significant differences in the bond order and local spin electronic structure indicators. To illustrate this, we have considered the electronic structure of representative molecular systems exhibiting different bonding situations, that is, the NaBH_3_^−^, BH_4_^−^ and NH_3_BH_3_. Relevant bond order, delocalization index, and local spin values (obtained in the framework of QTAIM)^[26]^ are gathered in Table 4.

**Table 4:** NBO and AIM charges (Q(E), E=H, NH_3_, Na), atomic spin density (*ρ*_s_), Wiberg Bond Order (WBO_NBO_), AIM Delocalization Index (DI_AIM_), Local Spins (⟨S^2^⟩), EDA Orbital Interaction (Δ*E*_orb_ in kcal mol^−1^) and electron density values at the E‐B (3,−1) point (*ρ*_BCP_) of BH_4_^−^, NH_3_BH_3_, and CS and BS NaBH_3_^−^ at PBE0/AVTZ and CASSCF/AVTZ.^[a,b]^

	BH_4_^−^	NH_3_BH_3_	NaBH_3_^−^ (CS)	NaBH_3_^−^ (BS)
**PBE0/AVTZ**				
Q(E)_NBO_ Q(B)_NBO_	−0.06 −0.76	+0.37 −0.23	−0.30 −0.45	−0.19 −0.56
Q(E)_AIM_ Q(B)_AIM_	−0.67 +1.70	+0.08 +1.84	−0.25 +1.52	−0.17 +1.43
WBO_NBO_ DI_AIM_	1.00 0.55	0.65 0.34	0.91 0.43	0.52 0.29
*ρ*_s_(E)_NBO_ *ρ*_s_(E)_AIM_	–	–	–	+0.66 +0.61
⟨S^2^⟩_E_/⟨S^2^⟩_B_	–	–	–	0.42/0.21
ρ_BCP_	0.15	0.11	0.013	0.014
Δ*E*_orb_(E^.^ + ^.^B)	−106.3	−263.3	−16.6	−19.2
Δ*E*_orb_(E:+B)	−154.2	−75.9	−34.8	−37.4
**CASSCF/AVTZ**				
Q(E)_AIM_ Q(B)_AIM_	−0.72 +1.91	+0.08 +2.04	−0.16 +1.61	
DI_AIM_	0.48	0.27	0.23	
⟨S^2^⟩_E_/⟨S^2^⟩_B_	0.02/0.02	0.03/0.03	0.33/0.26	
*ρ*_BCP_	0.15	0.10	0.015	
*c*_0_^[c]^	0.98	0.97	0.90	
*c*_d_^[c]^	−0.03	−0.05	−0.40

[a] On CCSD(T)/AVTZ structures. [b] See the Supporting Information for details. [c] CI coefficients.

Our minimal basis model explains the calculated Wiberg bond orders (WBO) in terms of the bond and spin polarization values, that can be easily derived from the NBO charges and spin populations (WBO_NBO_). For instance, for BH_4_^−^ *p*=0.06, so the expected bond order is 1−0.06^2^≈1. For NH_3_BH_3_, the donor NH_3_ unit has *δ*=0.37 and hence *p*=0.63, which would correspond to a bond order of 1−0.63^2^=0.60, in line with the computed WBO_NBO_=0.65. In the CS description of NaBH_3_^−^ *p*=0.30, leading to a bond order of 1−0.30^2^=0.91, in perfect agreement with the WBO_NBO_. In the BS case, the bond polarization is smaller (*p=*0.19) but there is significant spin polarization (*p_s_*=0.66), consistent with a bond order of 1−0.66^2^−0.19^2^=0.53, again in striking agreement with the exact WBO_NBO_.

For BH_4_^−^, the CASSCF(8,8) wave function displays a monodeterminantal character (*c*_0_=0.98 and *c_d_*=−0.03). WBO is 1.00, while the local spin values on B and H are negligible. Within the KS‐DFT description, the DI_AIM_ is somewhat smaller (0.55), driven by the large bond polarization produced by the QTAIM partitioning. EDA, QTAIM and NBO agree in an *electron‐sharing* picture as explained elsewhere.^[21]^ NH_3_BH_3_ is also well‐represented by one single‐determinant at CASSCF(12,12) (*c*_0_=0.97 and *c_d_*=−0.05). In this case, both the WBO_NBO_ and DI_AIM_ are smaller, as compared to the electron‐sharing case (0.65 and 0.34, respectively), but the local spin is again negligible. EDA delivers a lower orbital term for the fragmentation NH_3_(A_1_^2^) and BH_3_(A_1_^0^), so all indicators point towards a *dative* picture. Remarkably, the marked multi‐configurational character of NaBH_3_^−^, also captured by the BS solution, makes DI_AIM_ to drop to just 0.29, while the local spins on Na (0.42) and B (0.21) are now significant. The DI_AIM_ is significantly larger (0.43) for the CS solution, as the σ* contribution in the Na−B bond is absent. Note that a bonding analysis based on such a density would thus lead to inaccurately overestimated ionic interactions.^[22]^ In fact, the −0.50 Na Mulliken charge calculated with monoconfigurational DFT^[22]^ drops to −0.22 when switching to the CASSCF framework. The same trend is observed with the NBO and AIM charges for the CS and BS solutions. On the contrary, the BS solution mimics the CASSCF wave function, albeit with wrong spin symmetry (overall ⟨S^2^⟩=0.61). Both the DI_AIM_ and the local spin values are in good agreement with the CASSCF results. EDA favors Na(*s* ^1^) and BH_3_^−^(A_1_^1^) fragmentation in both the CS and BS solutions. Thus, combining bond orders and local spins analysis suggests that the Na‐B interaction in NaBH_3_^−^ is better described as a spin‐polarized bond, revealing its σ diradicaloid character.

To conclude, the exotic case of NaBH_3_^−^ cluster underscores the fundamental limitations of the conventional chemical bond classification into electron‐sharing and dative bonds. This binary Scheme remains useful for molecules like BH_4_^−^ or NH_3_BH_3_, which are well‐represented by a single‐determinant, but fails for multiconfigurational systems such as NaBH_3_^−^. Oversimplifying the wave function to a single CS configuration would essentially categorize a diradical as a conventional electron sharing bond. Within the KS‐DFT framework, the multi‐configurational character is partially recovered breaking the spin symmetry, allowing the localization of α and β electrons on distinct fragments. The assistance of other bonding indicators enables the identification of a third bonding category, namely a spin‐polarized bond, which captures the essence of the bonding in the NaBH_3_^−^ cluster.

## Conflict of interest

The authors declare no conflict of interest.

## Supporting information

As a service to our authors and readers, this journal provides supporting information supplied by the authors. Such materials are peer reviewed and may be re‐organized for online delivery, but are not copy‐edited or typeset. Technical support issues arising from supporting information (other than missing files) should be addressed to the authors.

Supplementary
