# Supplementary material for: Beyond the Classical Electron‐Sharing and Dative Bond Picture: Case of the Spin‐Polarized Bond
Source: Angew Chem Int Ed Engl. 2020 Nov 11;60(3):1498–502. doi: 10.1002/anie.202010948 (PMC7839703; doi:10.1002/anie.202010948)
Supplement: Supplementary file 1 — Supplementary [file ANIE-60-1498-s001.pdf]

Angewandte Chemie
Eine Zeitschrift der Gesellschaft Deutscher Chemiker

Supporting Information

Beyond the Classical Electron-Sharing and Dative Bond Picture: The Case of Spin-Polarized Bond

Pedro Salvador, Eva Vos, Inés Corral,* and Diego M. Andrada**

anie_202010948_sm_miscellaneous_information.pdf

Methods

All geometries were optimized with symmetry constraints within the DFT (density functional theory) framework using a series of functionals: BP86, [1] PBE,[2] B3LYP[3] TPSS,[4] M06L,[5] X3LYP,[6] PBE0,[2, 7] mPW1K,[8] BHHLYP,[9] TPPSh,[4, 10] M06,[11] M06-2X,[11] CAM-B3LYP,[12] wB97x,[13] B2PLYP,[14] mPW2PLYP,[15] PBE0DH,[16] and PBEQIDH.[17] The Dunning's basis set aug-cc-pVTZ (AVTZ) was selected for H, B, N, Na atoms.[18] These calculations were performed using the Gaussian 16 C01 software suite.[19] The stationary points were located with the Berny algorithm[20] using redundant internal coordinates. Analytical hessians were computed to determine the nature of stationary points[21] and to calculate thermal corrections and entropy effects using the standard statistical-mechanics relationships for an ideal gas.[22] The optimization at HF/AVTZ, MP2/AVTZ, CCSD/AVTZ, and CCSD(T)/AVTZ calculations were performed by employing MOLPRO 2019.1[23] software program package.

Multiconfigurational calculations for the lowest singlet and triplet states were undertaken using the SS-CASPT2//SS-CASPT2 protocol, that is SS-CASPT2 energies on SS-CASPT2 optimized geometries, as implemented the 1.2.2 version of Bagel.[24] SS-CASSCF wave functions with different active spaces were employed as a reference. The aug-cc-pVQZ (AVQZ) basis with the auxiliary cc-pVQZ-JKFIT basis set for density fitting were employed.

The reference CASSCF wave function for $NaBH_3^-$ includes 8 electrons in 8 orbitals as shown in Figure S1. The selected active space consists of the *3s* orbital of the Na, the *2s* and *2p* boron orbitals and the *1s* orbitals of the three hydrogen atoms. For BH_4^-, the active space, shown in Figure S2, contains the *2s* and the three *2p* orbitals of boron and the *1s* orbitals of each hydrogen atom. In contrast to the former systems, the (12,12) active space of NH_3BH_3 incorporates the *2s* and *2p* nitrogen orbitals, which replace the orbitals of Na, See Figure S3.

The CASPT2/aug-cc-pVQZ dissociation curve for $NaBH_3^-$, see Figure S4, was calculated using the external interface included in Gaussian16.[4] This interface allows performing relaxed scans using CASPT2 gradients, by linking Gaussian to Bagel. Interestingly, in contrast with monoconfigurational methods which only optimize the global C_s triplet minimum, CASPT2 is localizes an additional triplet minimum of C_{3v} symmetry, 7.7 kcal/mol over the singlet global minimum, underscoring the multireference character of this system.

The analysis of the CASSCF wave function along the dissociation curve reveals the multiconfigurational character of the system not only for significantly stretched Na-B bond

distances, but also at the equilibrium geometry. In fact, Truhlar's M diagnostic calculated half way to dissociation ($d_{Na\text{-}B}$=4.5 Å) and for the dissociated system ($d_{Na\text{-}B}$=7 Å) respectively amounts to 0.17 and 0.15, which correspond to strongly multiconfigurational situations according to the literature.[25] Even more correlated is the electronic structure of the system in the proximity of the equilibrium position ($d_{Na\text{-}B}$=2.75 Å) where M was predicted to amount to M=0.32.

For the calculation of bond orders and electronic spin calculations, single-point CASSCF/AVTZ calculations on the $NaBH_3^-$, BH_4^- and NH_3BH_3 CCSD(T)/AVTZ optimized structures were performed with pySCF.[26] The first- and second-order reduced density matrices necessary for the calculation of the local spin have been processed with APOST3D.[27] For BH_4^-, and $NaBH_3^-$, we used the (8,8) active spaces described above and in Figures S1-S2. For NH_3BH_3, an active space of 12 electrons in 12 orbitals has been used, including eight E orbitals and eight electrons associated to the B-H and N-H bonds, and four A_1 orbitals and four electrons for the B-N interaction.

The energy decomposition analysis (EDA) with the natural orbitals for chemical valence (NOCV) calculations on the optimized CCSD(T)/AVTZ geometries were carried out with ADF 2019.103.[28] The basis sets for all elements have quadruple-ζ quality augmented by four sets of polarizations functions. The level of theory used were BP86/QZ4P, M06L/QZ4P, and PBE0/QZ4P.[29] Scalar relativistic effects have been incorporated by applying the zeroth-order regular approximation (ZORA).[30]

Table S1. Triplet (ΔE_T) and BS (ΔE_{BS}) electronic energies relative to the CS solution (in kcal/mol) and Na-B equilibrium distance (R_e in Å) for singlet (CS), triplet (T), and singlet open shell (BS) states of $NaBH_3^-$ at different levels of theory. <S^2> and diradical character (n_{rad}).[a]

Method[b]		*CS* (*C_{3v}*)	*T* (*C_s*)		*BS* (*C_{3v}*)			
		R_e	ΔE_T	R_e	ΔE_{BS}	R_e	<S^2>	n_{rad} (%)
HF		4.865	−7.4	2.557	−7.8	2.797	0.89	67
MP2		2.763	−3.9	2.581	−4.8	2.710	0.90	68[c]
CCSD		2.794	1.6	2.574	−0.7	2.742	0.90	68[c]
CCSD(T)		2.719	5.2	2.580				0.14[d]
CASSCF(8,8)		2.862	5.0	2.556				56[e]
CASPT2(8,8)		2.666	4.3	2.552				56[e]
BP86	GGA	2.707	6.3	2.579	−0.4	2.702	0.30	16
PBE	GGA	2.690	6.0	2.562	-0.6	2.6801	0.35	19
TPSS	meta-GGA	2.757	5.3	2.553	−1.4	2.726	0.47	27
M06L	meta-GGA	2.699	1.5	2.482	−2.9	2.668	0.71	46
B3LYP	Hybrid GGA	2.763	6.3	2.565	−0.5	2.736	0.35	19
X3LYP	Hybrid GGA	2.759	6.4	2.557	−0.5	2.730	0.35	19
PBE0	Hybrid GGA	2.743	1.7	2.536	−2.8	2.681	0.61	38
mPW1K	Hybrid GGA	2.760	1.1	2.543	−3.1	2.692	0.63	39
BHHLYP	Hybrid GGA	2.862	2.7	2.533	−2.3	2.729	0.59	36
TPPSh	Hybrid mGGA	2.774	3.9	2.543	−2.1	2.723	0.54	32
M06	Hybrid mGGA	2.811	5.6	2.547	-0.4	2.722	0.43	25
M06-2X	Hybrid mGGA	2.698	3.9	2.536	-3.8	2.701	0.55	33
CAM-B3LYP	RS hybrid	2.745	2.1	2.529	-2.9	2.674	0.61	38
wB97x	RS hybrid	2.828	0.8	2.515	-3.7	2.668	0.63	39
B2PLYP	Double Hybrid	2.753	−0.4	2.562	−4.1	2.732	0.70	45
mPW2PLYP	Double Hybrid	2.759	0.0	2.553	−3.9	2.722	0.69	44
PBE0DH	Double Hybrid	2.767	−2.8	2.528	−5.7	2.679	0.75	50
PBEQIDH	Double Hybrid	2.766	−6.4	2.535	−8.2	2.683	0.89	67

[a] computed from <S^2> as described in [31].

[b] in combination with the AVTZ, except for CASSCF where the AVQZ was employed.

[c] from <S^2> of the HF wavefunction.

[d] largest t_2 amplitude.

[e] derived from the CI coefficient of the doubly-excited configuration.

Table S2. Electronic energies (in Hartrees) for singlet (CS), triplet (T), and singlet open shell (BS) states of $NaBH_3^-$.

Method	*CS (C_{3v})*	*T (C_s)*	*BS (C_{3v})*
HF/aug-cc-pVTZ	-188.2556577	-188.2674803	-188.2680566
MP2/aug-cc-pVTZ	-188.4014534	-188.4075964	-188.4090271
CCSD/aug-cc-pVTZ	-188.4323077	-188.42972	-188.4333956
CCSD(T)/aug-cc-pVTZ	-188.4430112	-188.4347851	--
BP86/aug-cc-pVTZ	-188.9523477	-188.9422874	-188.9529793
PBE/aug-cc-pVTZ	-188.7750556	-188.7655621	-188.7759658
TPSS/aug-ccpVTZ	-188.9702435	-188.9618539	-188.9724599
M06L/aug-cc-pVTZ	-188.9195959	-188.9172064	-188.9241653
B3LYP/aug-cc-pVTZ	-188.9688797	-188.9588047	-188.9697395
X3LYP/aug-cc-pVTZ	-188.9126212	-188.9023772	-188.9134973
PBE0/aug-cc-pVTZ	-188.7930286	-188.7903253	-188.7974385
mPW1K/aug-cc-pVTZ	-188.9241779	-188.9224379	-188.9290925
BHHLYP/aug-cc-pVTZ	-188.9085799	-188.9043136	-188.9122205
TPPSh/aug-cc-pVTZ	-188.958564	-188.9522839	-188.9619671
M06/aug-cc-pVTZ	-188.9013114	-188.8923961	-188.9019466
M06-2X/aug-cc-pVTZ	-188.892193	-188.8859512	-188.8981801
CAM-B3LYP/aug-cc-pVTZ	-188.9049954	-188.9016208	-188.90965
wB97x/aug-cc-pVTZ	-188.90736	-188.9060982	-188.9131892
B2PLYP/aug-cc-pVTZ	-188.7304605	-188.7311592	-188.7369521
mPW2PLYP/aug-cc-pVTZ	-188.74344	-188.7434972	-188.7496474
PBE0DH/aug-cc-pVTZ	-188.7368426	-188.7413625	-188.7458625
PBEQIDH/aug-cc-pVTZ	-188.6259554	-188.636177	-188.6389847

Table S3. CASSCF Configuration Interactions (CI) vector computed for BH_4^-, NH_3BH_3, and $NaBH_3^-$ at the equilibrium structure.

BH_4^-		NH_3BH_3		$NaBH_3^-$	
Configuration	CI	Configuration	CI	Configuration	CI
(22220000)	0.9804	(222222000000)	-0.9714	(22220000)	0.9009
(22200200)	-0.0594	(202222200000)	0.0506	(22202000)	-0.3963
(20220002)	-0.0558			(22020020)	-0.0522
(22020020)	-0.0505			(20220002)	-0.0522

Table S4. NBO and AIM charges (Q (E), E = H, NH_3, Na), atomic spin density (ρ_s), Wiberg Bond Order (WBO_{NBO}), AIM Delocalization Index (DI_{AIM}), Local Spins (<S^2>), EDA Orbital Interaction (ΔE_{orb} in kcal/mol) and electron density values at the E-B (3,-1) point (ρ_{BCP}) of BH_4^-, NH_3BH_3, and CS and BS $NaBH_3^-$ at BP86/AVTZ.[a]

	BH_4^-	NH_3BH_3	$NaBH_3^-$ (CS)	$NaBH_3^-$ (BS)
$Q(E)_{NBO}$/ $Q(B)_{NBO}$	-0.05/-0.79	+0.38/-0.25	-0.25/-0.51	-0.23/-0.54
$\rho_s(E)_{NBO}$ / $\rho_s(E)_{AIM}$				0.46 / 0.42
$Q(E)_{AIM}$/ $Q(B)_{AIM}$	-0.65/1.61	0.08/1.76	-0.22/1.39	-0.20/1.36
ρ_{BCP}	0.15	0.11	0.014	0.014
WBO_{NBO} / DI_{AIM}	1.00/0.58	0.66/0.36	0.93/0.47	0.73/0.39
$\langle S^2\rangle_E/\langle S^2\rangle_B$				0.20/0.11
ΔE_{orb}(E• + •B)	−100.7	−257.9	−18.9	−19.5
ΔE_{orb}(E: + B)	−154.7	−77.9	−39.0	−39.6

[a] on CCSD(T)/AVTZ structures.

Table S5. NBO and AIM charges (Q (E), E = H, NH_3, Na), atomic spin density (ρ_s), Wiberg Bond Order (WBO_{NBO}), AIM Delocalization Index (DI_{AIM}), Local Spins (<S^2>), EDA Orbital Interaction (ΔE_{orb} in kcal/mol), and electron density values at the E-B (3,-1) point (ρ_{BCP}) of BH_4^-, NH_3BH_3, and CS and BS $NaBH_3^-$ at M06L/AVTZ.[a]

	BH_4^-	NH_3BH_3	$NaBH_3^-$ (CS)	$NaBH_3^-$ (BS)
$Q(E)_{NBO/}$ $Q(B)_{NBO}$	-0.09/-0.65	0.36/-0.15	-0.31/-0.38	-0.18/-0.49
$\rho_s(E)_{NBO}$ / $\rho_s(E)_{AIM}$				0.68 / 0.67
$Q(E)_{AIM}$/ $Q(B)_{AIM}$	-0.70/1.78	0.08/1.88	-0.26/1.54	-0.20/1.46
ρ	0.15	0.10	0.013	0.013
WBO_{NBO} / DI_{AIM}	0.99/0.53	0.64/0.33	0.90/0.43	0.49/0.25
$\langle S^2\rangle_E/\langle S^2\rangle_B$				0.50/0.27
ΔE_{orb}(E• + •B)	−106.6	−270.0	−22.1	−24.8
ΔE_{orb}(E: + B)	−153.2	−70.4	−38.2	−41.0

[a] on CCSD(T)/AVTZ structures.

Table S6. Energy decomposition analysis (EDA) of $NaBH_3^-$ (CS), $NaBH_3^-$ (BS), BH_4^- and NH_3BH_3 at the BP86/QZ4P level of theory.[a] Energy values are given in kcal/mol.

	$(NaBH_3)^-$ Singlet closed-shell		
	$Na^-(s^2)$; BH_3 (A_1^0)	$Na(s^1)$; BH_3^- (A_1^1)	$Na^+(s^0)$; BH_3^{2-} (A_1^2)
ΔE_{int}	-22.3	-30.7	-280.9
ΔE_{Pauli}	33.7	28.6	67.3
$\Delta E_{elstat}{}^b$	-17.0 (30.4%)	-40.5(68.2%)	-287.7(82.6%)
$\Delta E_{orb}{}^b$	-39.0 (69.6%)	***-18.9*** (31.8%)	-60.5(17.4%)
ΔE_{prep}	1.2	9.6	259.9
$D_e{}^{c,d}$	21.1	21.1	21.1
	$(NaBH_3)^-$ Broken Symmetry		
	$Na^-(s^2)$; BH_3 (A_1^0)	$Na(s^1)$; BH_3^- (A_1^1)	$Na^+(s^0)$; BH_3^{2-} (A_1^2)
ΔE_{int}	-22.9	-31.3	-281.6
ΔE_{Pauli}	33.7	28.6	67.3
$\Delta E_{elstat}{}^b$	-17.0(30.0%)	-40.5(67.5%)	-287.7(82.5%)
$\Delta E_{orb}{}^b$	-39.6(70.0%)	***-19.5***(32.5%)	-61.1(17.5%)
ΔE_{prep}	1.2	9.6	259.9
$D_e{}^{c,d}$	21.7	21.7	21.7
	BH_4^-		
	$H^-(s^2)$; BH_3 (A_1^0)	$H(s^1)$; BH_3^- (A_1^1)	$H^+(s^0)$; BH_3^{2-} (A_1^2)
ΔE_{int}	-101.7	-102.6	-535.7
ΔE_{Pauli}	137.9	39.2	0.0
$\Delta E_{elstat}{}^b$	-84.9(35.4%)	-41.1(29.0%)	-245.8(45.9%)
$\Delta E_{orb}{}^b$	-154.7(64.6%)	***-100.7***(71.0%)	-289.9(54.1%)
ΔE_{prep}	22.2	23.1	456.2
$D_e{}^{c,d}$	79.5	79.5	79.5
	$H_3N–BH_3$		
	NH_3 ($A1^2$); BH_3 (A_1^0)	NH_3^+ (A_1^1); BH_3^- (A_1^1)	NH_3^{2+}(A_1^0); BH_3^{2-} (A_1^2)
ΔE_{int}	-44.6	-284.9	-988.3
ΔE_{Pauli}	110.3	200.0	312.9
$\Delta E_{elstat}{}^b$	-77.0(49.7%)	-227.0(46.8%)	-618.3(47.5%)
$\Delta E_{orb}{}^b$	***-77.9***(50.3%)	-257.9(53.2%)	-682.9(52.5%)
ΔE_{prep}	12.3	252.5	956.0
$D_e{}^{c,d}$	32.4	32.4	32.4

[a] All calculations were performed on the CCSD(T)/aug-cc-pVTZ optimized structures. [b] The value in parenthesis gives the percentage contribution to the total attractive interactions ΔE_{elstat} + ΔE_{orb}. [c] The dissociation energy (D_e) has been computed at the specific electronic state. [d] computed as a single point the CCSD(T)/aug-cc-pVTZ optimized structure.

Table S7. Energy decomposition analysis (EDA) of $NaBH_3^-$ (CS), $NaBH_3^-$ (BS), BH_4^- and NH_3BH_3 at the M06L/QZ4P level of theory.[a] Energy values are given in kcal/mol.

	$(NaBH_3)^-$ Singlet closed-shell		
	$Na^-(s^2)$; BH_3 (A_1^0)	$Na(s^1)$; BH_3^- (A_1^1)	$Na^+(s^0)$; BH_3^{2-} (A_1^2)
ΔE_{int}	-17.9	-37.9	-269.9
ΔE_{Pauli}	36.5	31.9	75.4
ΔE_{elstat}^b	-16.1 (29.7%)	-47.8(68.4%)	-286.7(83.0%)
ΔE_{orb}^b	-38.2(70.3%)	***-22.1***(31.6%)	-58.6(17.0%)
ΔE_{prep}	1.6	21.7	253.7
$D_e^{c,d}$	16.2	16.2	16.2
	$(NaBH_3)^-$ Broken Symmetry		
	$Na^-(s^2)$; BH_3 (A_1^0)	$Na(s^1)$; BH_3^- (A_1^1)	$Na^+(s^0)$; BH_3^{2-} (A_1^2)
ΔE_{int}	-20.6	-40.7	-272.7
ΔE_{Pauli}	36.5	31.9	75.4
ΔE_{elstat}^b	-16.1(28.2%)	-47.8(65.8%)	-286.7(82.4%)
ΔE_{orb}^b	-41.0(71.8%)	***-24.8***(34.2%)	-61.3(17.6%)
ΔE_{prep}	1.6	21.7	253.7
$D_e^{c,d}$	19.0	19.0	19.0
	BH_4^-		
	$H^-(s^2)$; BH_3 (A_1^0)	$H(s^1)$; BH_3^- (A_1^1)	$H^+(s^0)$; BH_3^{2-} (A_1^2)
ΔE_{int}	-101.6	-106.1	-546.1
ΔE_{Pauli}	136.6	41.4	0.0
ΔE_{elstat}^b	-85.0(35.7%)	-40.8(27.7%)	-246.9(45.2%)
ΔE_{orb}^b	-153.2(64.3%)	**-106.6**(72.3%)	-299.2(54.8%)
ΔE_{prep}	23.5	28.0	468.0
$D_e^{c,d}$	78.1	78.1	78.1
	$H_3N–BH_3$		
	NH_3 $(A1^2)$; BH_3 (A_1^0)	NH_3^+ (A_1^1); BH_3^- (A_1^1)	$NH_3^{2+}(A_1^0)$; BH_3^{2-} (A_1^2)
ΔE_{int}	-45.1	-290.6	-991.0
ΔE_{Pauli}	102.0	204.7	317.3
ΔE_{elstat}^b	-76.6(52.1%)	-225.3(45.5%)	-614.3(47.0%)
ΔE_{orb}^b	***-70.4***(47.9%)	-270.0(54.5%)	-694.0(53.0%)
ΔE_{prep}	12.8	258.4	958.7
$D_e^{c,d}$	32.2	32.2	32.2

[a] All calculations were performed on the CCSD(T)/aug-cc-pVTZ optimized structures. [b] The value in parenthesis gives the percentage contribution to the total attractive interactions ΔE_{elstat} + ΔE_{orb}. [c] the dissociation energy (D_e) has been computed at the specific electronic state. [d] computed as a single point the CCSD(T)/aug-cc-pVTZ optimized structure.

Table S8. Energy decomposition analysis (EDA) of $NaBH_3^-$ (CS), $NaBH_3^-$ (BS), BH_4^- and NH_3BH_3 at the PBE0/QZ4P level of theory.[a] Energy values are given in kcal/mol.

	$(NaBH_3)^-$ Singlet closed-shell		
	$Na^-(s^2)$; BH_3 (A_1^0)	$Na(s^1)$; BH_3^- (A_1^1)	$Na^+(s^0)$; BH_3^{2-} (A_1^2)
ΔE_{int}	-18.5	-29.5	-283.1
ΔE_{Pauli}	33.4	30.7	66.6
ΔE_{elstat}^b	-17.1 (32.9%)	-43.6(72.4%)	-289.6(82.8%)
ΔE_{orb}^b	-34.8 (67.1%)	***-16.6***(27.6%)	-60.0(17.2%)
ΔE_{prep}	1.4	12.3	265.9
$D_e^{c,d}$	17.1	17.1	17.1
	$(NaBH_3)^-$ Broken Symmetry		
	$Na^-(s^2)$; BH_3 (A_1^0)	$Na(s^1)$; BH_3^- (A_1^1)	$Na^+(s^0)$; BH_3^{2-} (A_1^2)
ΔE_{int}	-21.1	-32.1	-285.7
ΔE_{Pauli}	33.4	30.7	66.6
ΔE_{elstat}^b	-17.1(31.4%)	-43.6(69.4%)	-289.6(82.2%)
ΔE_{orb}^b	-37.4(68.6%)	***-19.2***(30.6%)	-62.7(17.8%)
ΔE_{prep}	1.4	12.3	266.0
$D_e^{c,d}$	19.8	19.8	19.8
	BH_4^-		
	$H^-(s^2)$; BH_3 (A_1^0)	$H(s^1)$; BH_3^- (A_1^1)	$H^+(s^0)$; BH_3^{2-} (A_1^2)
ΔE_{int}	-104.8	-103.0	-540.4
ΔE_{Pauli}	136.3	42.6	0.0
ΔE_{elstat}^b	-86.9(36.0%)	-39.3(27.0%)	-247.5(45.8%)
ΔE_{orb}^b	-154.2(64.0%)	***-106.3***(73.0%)	-293.0(54.2%)
ΔE_{prep}	23.2	21.4	458.8
$D_e^{c,d}$	81.6	81.6	81.6
	$H_3N–BH_3$		
	NH_3 ($A1^2$); BH_3 (A_1^0)	NH_3^+ (A_1^1); BH_3^- (A_1^1)	NH_3^{2+}(A_1^0); BH_3^{2-} (A_1^2)
ΔE_{int}	-47.3	-288.8	-993.9
ΔE_{Pauli}	105.4	202.3	317.9
ΔE_{elstat}^b	-76.8(50.3%)	-227.8(46.4%)	-625.3(47.7%)
ΔE_{orb}^b	***-75.9***(49.7%)	-263.3(53.6%)	-686.6(52.3%)
ΔE_{prep}	12.7	254.2	959.4
$D_e^{c,d}$	34.5	34.5	34.5

[a] All calculations were performed on the CCSD(T)/aug-cc-pVTZ optimized structures. [b] The value in parenthesis gives the percentage contribution to the total attractive interactions ΔE_{elstat} + ΔE_{orb}. [c] the dissociation energy (D_e) has been computed at the specific electronic state. [d] computed as a single point the CCSD(T)/aug-cc-pVTZ optimized structure.

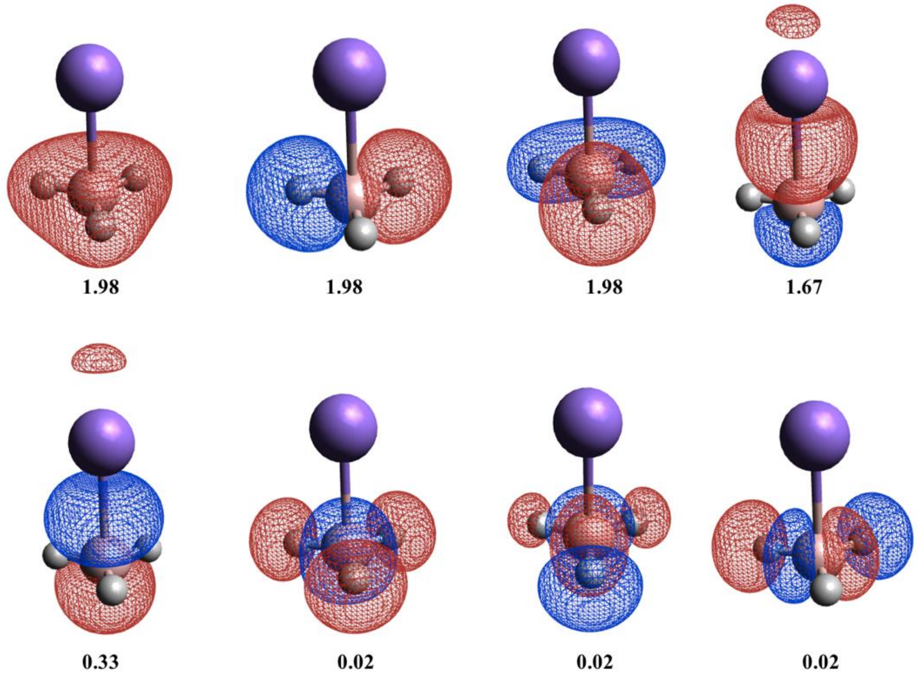

Figure S1. (8,8) active space for $NaBH_3^-$ at the CASPT2/AVQZ equilibrium geometry, including the orbital occupation numbers.

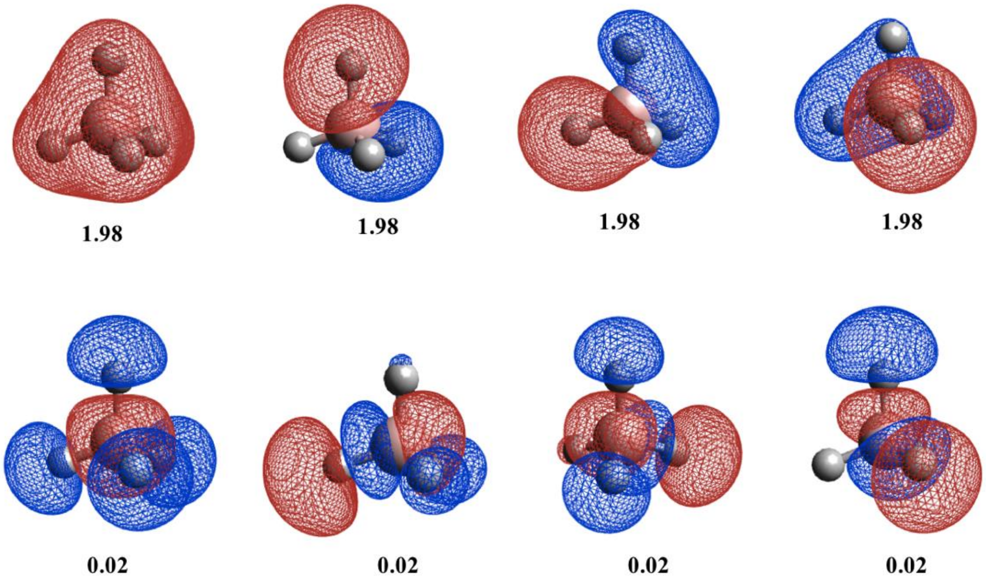

Figure S2. (8,8) active space for BH_4^- at the CASPT2/AVQZ equilibrium geometry, including the orbital occupation numbers.

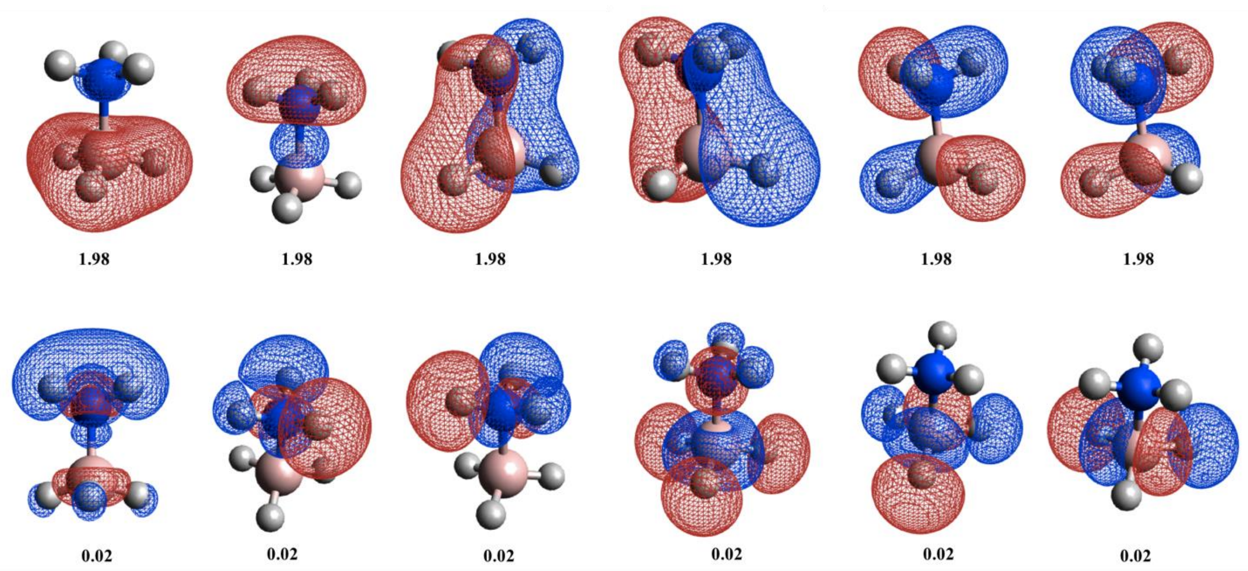

Figure S3. (12,12) active space for NH_3BH_3 system at the CASPT2/AVQZ equilibrium geometry, including the orbital occupation numbers.

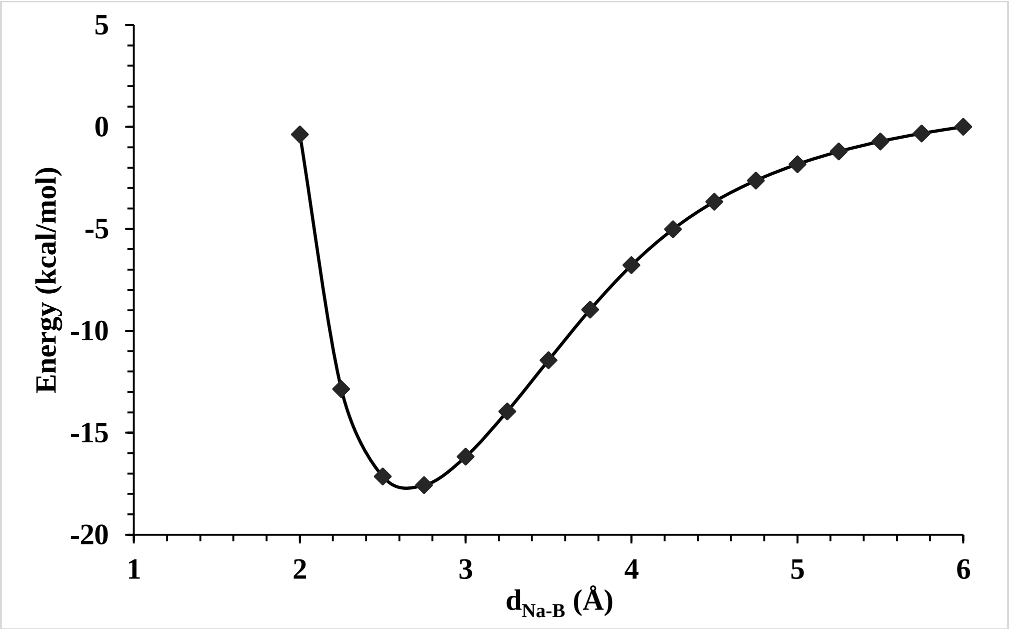

Figure S4. $NaBH_3^-$ dissociation curve calculated at the CASPT2/AVQZ level of theory

XYZ coordinates and Absolute Energies in Hartrees

(NaBH$_3$)$^-$ (singlet)
HF/aug-cc-pVTZ= -188.255657746

B	0.000000000000	0.000000000000	-2.812690000000
H	0.000000000000	1.187973000000	-2.837327000000
H	1.028815000000	-0.593987000000	-2.837327000000
H	-1.028815000000	-0.593987000000	-2.837327000000
Na	0.000000000000	0.000000000000	2.052312000000

(NaBH$_3$)$^-$ (singlet open shell)
HF/aug-cc-pVTZ= -188.268056636

B	0.000000000000	0.000000000000	-1.596607000000
H	0.000000000000	1.199085000000	-1.740075000000
H	1.038438000000	-0.599543000000	-1.740075000000
H	-1.038438000000	-0.599543000000	-1.740075000000
Na	0.000000000000	0.000000000000	1.200297000000

(NaBH$_3$)$^-$ (triplet)
HF/aug-cc-pVTZ= -188.267480345

Na	-0.021656000000	-1.083543000000	0.000000000000
B	-0.021656000000	1.473574000000	0.000000000000
H	-0.342176000000	2.627918000000	0.000000000000
H	0.344332000000	0.961593000000	1.039337000000
H	0.344332000000	0.961593000000	-1.039337000000

(NaBH$_3$)$^-$ (singlet)
MP2/aug-cc-pVTZ= -188.247574245

B	0.000000000000	0.000000000000	-1.580051000000
H	0.000000000000	1.197491000000	-1.702947000000
H	1.037058000000	-0.598746000000	-1.702947000000
H	-1.037058000000	-0.598746000000	-1.702947000000
Na	0.000000000000	0.000000000000	1.182645000000

(NaBH$_3$)$^-$ (singlet open shell)
MP2/aug-cc-pVTZ= -188.267603805

B	0.000000000000	0.000000000000	-1.557213000000
H	0.000000000000	1.202916000000	-1.631632000000
H	1.041755000000	-0.601458000000	-1.631632000000
H	-1.041755000000	-0.601458000000	-1.631632000000
Na	0.000000000000	0.000000000000	1.152815000000

(NaBH$_3$)$^-$ (triplet)
MP2/aug-cc-pVTZ= -188.267176170

Na	-0.009453000000	-1.088197000000	0.000000000000
B	-0.009453000000	1.492507000000	0.000000000000
H	-0.243241000000	2.666061000000	0.000000000000
H	0.197247000000	0.920785000000	1.046184000000
H	0.197247000000	0.920785000000	-1.046184000000

(NaBH$_3$)$^-$ (singlet)
CCSD/aug-cc-pVTZ= -188.4323077

B	0.000000000000	0.000000000000	-1.601178000000
H	0.000000000000	1.200697000000	-1.704129000000
H	1.039834000000	-0.600348000000	-1.704129000000
H	-1.039834000000	-0.600348000000	-1.704129000000
Na	0.000000000000	0.000000000000	1.192571000000

(NaBH$_3$)$^-$ (singlet open shell)
CCSD/aug-cc-pVTZ= -188.4333956

B	0.000000000000	0.000000000000	-1.571274000000
H	0.000000000000	1.203324000000	-1.674596000000
H	1.042109000000	-0.601662000000	-1.674596000000
H	-1.042109000000	-0.601662000000	-1.674596000000
Na	0.000000000000	0.000000000000	1.170923000000

(NaBH$_3$)$^-$ (triplet)
CCSD/aug-cc-pVTZ= -188.429702

Na	-0.011999000000	-1.086143000000	0.000000000000
B	-0.011999000000	1.487548000000	0.000000000000
H	-0.274075000000	2.658532000000	0.000000000000
H	0.233033000000	0.925648000000	1.048033000000
H	0.233033000000	0.925648000000	-1.048033000000

(NaBH$_3$)$^-$ (singlet)
CCSD(T)/aug-cc-pVTZ=-188.44301121

B	0.000000000000	-0.000000245100	-1.589247392800
H	0.000000000000	1.206137827800	-1.682180509100
H	1.044546213800	-0.603069269600	-1.682180378700
H	-1.044546213800	-0.603069269600	-1.682180378700
Na	0.000000000000	-0.000000043500	1.129541659200

(NaBH$_3$)$^-$ (triplet)
CCSD(T)/aug-cc-pVTZ=-188.43478510

Na	0.0496436337	-1.0943927105	0.0000000000
B	-0.0370246199	1.4838028170	0.0000000000
H	-0.3306571828	2.6483968447	0.0000000000
H	0.2174655845	0.9262575244	1.0494489804
H	0.2174655845	0.9262575244	-1.0494489804

(NaBH$_3$)$^-$ (singlet)
BP86/aug-cc-pVTZ= -188.952347699

B	0.000000000000	0.000000000000	-1.553490000000
H	0.000000000000	1.208141000000	-1.638662000000
H	1.046281000000	-0.604071000000	-1.638662000000
H	-1.046281000000	-0.604071000000	-1.638662000000
Na	0.000000000000	0.000000000000	1.153040000000

(NaBH$_3$)$^-$ (singlet open shell)
BP86/aug-cc-pVTZ= -188.952979279

B	0.000000000000	0.000000000000	-1.551542000000
H	0.000000000000	1.209313000000	-1.633603000000
H	1.047295000000	-0.604656000000	-1.633603000000
H	-1.047295000000	-0.604656000000	-1.633603000000
Na	0.000000000000	0.000000000000	1.150774000000

(NaBH$_3$)$^-$ (triplet)
BP86/aug-cc-pVTZ= -188.942287421

Na	-0.008600000000	-1.089070000000	0.000000000000
B	-0.008600000000	1.489818000000	0.000000000000
H	-0.500007000000	2.591726000000	0.000000000000
H	0.318805000000	0.969478000000	1.053217000000
H	0.318805000000	0.969478000000	-1.053217000000

(NaBH$_3$)$^-$ (singlet)
PBE/aug-cc-pVTZ= -188.775055645

B	0.000000000000	0.000000000000	-1.543924000000
H	0.000000000000	1.208684000000	-1.629550000000
H	1.046751000000	-0.604342000000	-1.629550000000
H	-1.046751000000	-0.604342000000	-1.629550000000
Na	0.000000000000	0.000000000000	1.146206000000

(NaBH$_3$)$^-$ (singlet open shell)
PBE/aug-cc-pVTZ=-188.775965862

B	0.000000000000	-0.000019000000	-1.627476000000
H	-0.000001000000	1.210083000000	-1.708563000000
H	1.047971000000	-0.605059000000	-1.708707000000
H	-1.047971000000	-0.605058000000	-1.708709000000
Na	0.000001000000	0.000054000000	1.053287000000

(NaBH$_3$)$^-$ (triplet)
PBE/aug-cc-pVTZ=-188.765562127

Na	-0.009068000000	-1.082205000000	0.000000000000
B	-0.009068000000	1.479608000000	0.000000000000
H	-0.506720000000	2.578855000000	0.000000000000
H	0.325904000000	0.963681000000	1.053817000000
H	0.325904000000	0.963681000000	-1.053817000000

$(NaBH_3)^-$ (singlet)
TPSS/aug-cc-pVTZ=-188.970243536

B	0.000000000000	0.000000000000	-1.583482000000
H	0.000000000000	1.202229000000	-1.662011000000
H	1.041161000000	-0.601115000000	-1.662011000000
H	-1.041161000000	-0.601115000000	-1.662011000000
Na	0.000000000000	0.000000000000	1.173040000000

$(NaBH_3)^-$ (singlet open shell)
TPSS/aug-cc-pVTZ=-188.972459940

B	0.000000000000	0.000000000000	-1.564878000000
H	0.000000000000	1.204985000000	-1.648745000000
H	1.043548000000	-0.602493000000	-1.648745000000
H	-1.043548000000	-0.602493000000	-1.648745000000
Na	0.000000000000	0.000000000000	1.160966000000

$(NaBH_3)^-$ (triplet)
TPSS/aug-cc-pVTZ=-188.961853875

Na	-0.012590000000	-1.079756000000	0.000000000000
B	-0.012590000000	1.473686000000	0.000000000000
H	-0.463574000000	2.587407000000	0.000000000000
H	0.332509000000	0.960742000000	1.047806000000
H	0.332509000000	0.960742000000	-1.047806000000

$(NaBH_3)^-$ (singlet)
M06L/aug-cc-pVTZ= -188.919595942

B	0.000000000000	0.000000000000	-1.551067000000
H	0.000000000000	1.194021000000	-1.623453000000
H	1.034052000000	-0.597010000000	-1.623453000000
H	-1.034052000000	-0.597010000000	-1.623453000000
Na	0.000000000000	0.000000000000	1.147790000000

$(NaBH_3)^-$ (singlet open shell)
M06L/aug-cc-pVTZ=-188.924165349

B	0.000000000000	0.000000000000	-1.530158000000
H	0.000000000000	1.195433000000	-1.623083000000
H	1.035275000000	-0.597716000000	-1.623083000000
H	-1.035275000000	-0.597716000000	-1.623083000000
Na	0.000000000000	0.000000000000	1.138185000000

$(NaBH_3)^-$ (triplet)
M06L/aug-cc-pVTZ= -188.917206402

Na	-1.055461000000	0.000064000000	-0.033939000000
B	1.426101000000	-0.000241000000	-0.006519000000
H	2.518085000000	0.005678000000	-0.485011000000
H	0.977460000000	1.028103000000	0.447928000000
H	0.984019000000	-1.033279000000	0.443003000000

$(NaBH_3)^-$ (singlet)
B3LYP/aug-cc-pVTZ= -188.968879662

B	0.000000000000	0.000000000000	-1.586308000000
H	0.000000000000	1.195921000000	-1.669715000000
H	1.035698000000	-0.597960000000	-1.669715000000
H	-1.035698000000	-0.597960000000	-1.669715000000
Na	0.000000000000	0.000000000000	1.176426000000

$(NaBH_3)^-$ (singlet open shell)
B3LYP/aug-cc-pVTZ= -188.969739549

B	0.000000000000	0.000000000000	-1.570656000000
H	0.000000000000	1.197637000000	-1.654967000000
H	1.037184000000	-0.598818000000	-1.654967000000
H	-1.037184000000	-0.598818000000	-1.654967000000
Na	0.000000000000	0.000000000000	1.165289000000

$(NaBH_3)^-$ (triplet)
B3LYP/aug-cc-pVTZ= -188.958804675

Na	-0.008350000000	-1.082046000000	0.000000000000
B	-0.008350000000	1.482535000000	0.000000000000
H	-0.397345000000	2.613375000000	0.000000000000
H	0.265469000000	0.938229000000	1.043631000000
H	0.265469000000	0.938229000000	-1.043631000000BHH

$(NaBH_3)^-$ (singlet)
X3LYP/aug-cc-pVTZ=-188.912621196

B	0.000000000000	0.000000000000	-1.584068000000
H	0.000000000000	1.195645000000	-1.666543000000
H	1.035459000000	-0.597823000000	-1.666543000000
H	-1.035459000000	-0.597823000000	-1.666543000000
Na	0.000000000000	0.000000000000	1.174542000000

$(NaBH_3)^-$ (singlet open shell)
X3YP/aug-cc-pVTZ=-188.913497292

B	0.000000000000	0.000000000000	-1.567137000000
H	0.000000000000	1.197395000000	-1.650530000000
H	1.036975000000	-0.598698000000	-1.650530000000
H	-1.036975000000	-0.598698000000	-1.650530000000
Na	0.000000000000	0.000000000000	1.162480000000

$(NaBH_3)^-$ (triplet)
X3LYP/aug-cc-pVTZ=-188.902377233

Na	-0.008190000000	-1.078795000000	0.000000000000
B	-0.008190000000	1.478518000000	0.000000000000
H	-0.379470000000	2.614922000000	0.000000000000
H	0.255253000000	0.929615000000	1.043641000000
H	0.255253000000	0.929615000000	-1.043641000000

$(NaBH_3)^-$ (singlet)
PBE0/aug-cc-pVTZ= -188.793028628

B	0.000000000000	0.000000000000	-1.574074000000
H	0.000000000000	1.201702000000	-1.662799000000
H	1.040704000000	-0.600851000000	-1.662799000000
H	-1.040704000000	-0.600851000000	-1.662799000000
Na	0.000000000000	0.000000000000	1.168979000000

$(NaBH_3)^-$ (singlet open shell)
PBE0/aug-cc-pVTZ= -188.797438526

B	0.000000000000	0.000000000000	-1.538493000000
H	0.000000000000	1.205151000000	-1.624918000000
H	1.043692000000	-0.602576000000	-1.624918000000
H	-1.043692000000	-0.602576000000	-1.624918000000
Na	0.000000000000	0.000000000000	1.142474000000

$(NaBH_3)^-$ (triplet)
PBE0/aug-cc-pVTZ= -188.790325344

Na	-0.012288000000	-1.072113000000	0.000000000000
B	-0.012288000000	1.463719000000	0.000000000000
H	-0.433692000000	2.588743000000	0.000000000000
H	0.315148000000	0.942952000000	1.048754000000
H	0.315148000000	0.942952000000	-1.048754000000

$(NaBH_3)^-$ (singlet)
mPW1PW91/aug-cc-pVTZ=-188.924177849

B	0.000000000000	0.000000000000	-1.583904000000
H	0.000000000000	1.199559000000	-1.672956000000
H	1.038848000000	-0.599779000000	-1.672956000000
H	-1.038848000000	-0.599779000000	-1.672956000000
Na	0.000000000000	0.000000000000	1.176217000000

$(NaBH_3)^-$ (singlet open shell)
mPW1PW91/aug-cc-pVTZ=-188.929092471

B	0.000000000000	0.000000000000	-1.544555000000
H	0.000000000000	1.203265000000	-1.633370000000
H	1.042058000000	-0.601632000000	-1.633370000000
H	-1.042058000000	-0.601632000000	-1.633370000000
Na	0.000000000000	0.000000000000	1.147535000000

$(NaBH_3)^-$ (triplet)
mPW1PW91/aug-cc-pVTZ=-188.922437935

Na	-0.012610000000	-1.075180000000	0.000000000000
B	-0.012610000000	1.467470000000	0.000000000000
H	-0.437112000000	2.589499000000	0.000000000000
H	0.319437000000	0.950067000000	1.046854000000
H	0.319437000000	0.950067000000	-1.046854000000

$(NaBH_3)^-$ (singlet)
BHHLYP/aug-cc-pVTZ= -188.908579872

B	0.000000000000	0.000000000000	-1.644270000000
H	0.000000000000	1.188300000000	-1.723742000000
H	1.029098000000	-0.594150000000	-1.723742000000
H	-1.029098000000	-0.594150000000	-1.723742000000
Na	0.000000000000	0.000000000000	1.217507000000

$(NaBH_3)^-$ (singlet open shell)
BHHLYP/aug-cc-pVTZ=-188.912220478

B	0.000000000000	0.000000000000	-1.565205000000
H	0.000000000000	1.192359000000	-1.657450000000
H	1.032613000000	-0.596179000000	-1.657450000000
H	-1.032613000000	-0.596179000000	-1.657450000000
Na	0.000000000000	0.000000000000	1.163489000000

$(NaBH_3)^-$ (triplet)
BHHLYP/aug-cc-pVTZ= -188.904313642

Na	-0.010903000000	-1.069052000000	0.000000000000
B	-0.010903000000	1.464182000000	0.000000000000
H	-0.315797000000	2.614490000000	0.000000000000
H	0.245120000000	0.912088000000	1.038613000000
H	0.245120000000	0.912088000000	-1.038613000000

$(NaBH_3)^-$ (singlet)
TPSSh/aug-cc-pVTZ=-188.958563962

B	0.000000000000	0.000000000000	-1.593381000000
H	0.000000000000	1.200127000000	-1.673824000000
H	1.039340000000	-0.600063000000	-1.673824000000
H	-1.039340000000	-0.600063000000	-1.673824000000
Na	0.000000000000	0.000000000000	1.180761000000

$(NaBH_3)^-$ (singlet open shell)
TPSSh/aug-cc-pVTZ=-188.961967066

B	0.000000000000	0.000000000000	-1.562722000000
H	0.000000000000	1.203453000000	-1.650654000000
H	1.042221000000	-0.601726000000	-1.650654000000
H	-1.042221000000	-0.601726000000	-1.650654000000
Na	0.000000000000	0.000000000000	1.160507000000

$(NaBH_3)^-$ (triplet)
TPSSh/aug-cc-pVTZ=-188.952283948

Na	-0.013897000000	-1.075723000000	0.000000000000
B	-0.013897000000	1.467169000000	0.000000000000
H	-0.443113000000	2.587972000000	0.000000000000
H	0.332735000000	0.954569000000	1.046202000000
H	0.332735000000	0.954569000000	-1.046202000000

$(NaBH_3)^-$ (singlet)
M06/aug-cc-pVTZ= -188.901311361

B	1.618301000000	-0.000005000000	-0.000011000000
H	1.676527000000	0.862915000000	-0.826120000000
H	1.676805000000	0.283959000000	1.160328000000
H	1.676442000000	-1.146910000000	-0.334277000000
Na	-1.192844000000	0.000006000000	0.000011000000

$(NaBH_3)^-$ (singlet open shell)
M06/aug-cc-pVTZ= -188.901946682

B	-0.000002000000	-0.000032000000	-1.638342000000
H	0.000002000000	1.196114000000	-1.715007000000
H	1.035868000000	-0.598097000000	-1.715287000000
H	-1.035869000000	-0.598100000000	-1.715289000000
Na	0.000001000000	0.000115000000	1.083756000000

$(NaBH_3)^-$ (triplet)
M06/aug-cc-pVTZ= -188.892396149

Na	-0.014704000000	-1.077169000000	0.000000000000
B	-0.014704000000	1.469482000000	0.000000000000
H	-0.400982000000	2.598083000000	0.000000000000
H	0.318124000000	0.951681000000	1.040403000000
H	0.318124000000	0.951681000000	-1.040403000000

$(NaBH_3)^-$ (singlet)
M06-2X/aug-cc-pVTZ=-188.892192983

B	1.545500000000	0.000045000000	-0.000077000000
H	1.651764000000	-1.091564000000	0.486465000000
H	1.648094000000	0.124456000000	-1.189041000000
H	1.650296000000	0.967559000000	0.701757000000
Na	-1.152514000000	-0.000062000000	0.000109000000

$(NaBH_3)^-$ (singlet open shell)
M06-2X/aug-cc-pVTZ= -188.898180152

B	0.000000000000	0.000000000000	-1.679853000000
H	0.000000000000	1.206138000000	-1.772786000000
H	1.044546000000	-0.603069000000	-1.772786000000
H	-1.044546000000	-0.603069000000	-1.772786000000
Na	0.000000000000	0.000000000000	1.220147000000

$(NaBH_3)^-$ (triplet)
M06-2X/aug-cc-pVTZ=-188.885951207

Na	0.283041000000	-1.034700000000	0.000000000000
B	-0.023819000000	1.482668000000	0.000000000000
H	-0.167410000000	2.667884000000	0.000000000000
H	0.047811000000	0.891063000000	1.048369000000
H	0.047811000000	0.891063000000	-1.048369000000

$(NaBH_3)^-$ (singlet)
CAM-B3LYP/aug-cc-pVTZ= -188.904995414

B	0.000000000000	0.000000000000	-1.573466000000
H	0.000000000000	1.193014000000	-1.674330000000
H	1.033181000000	-0.596507000000	-1.674330000000
H	-1.033181000000	-0.596507000000	-1.674330000000
Na	0.000000000000	0.000000000000	1.171847000000

$(NaBH_3)^-$ (singlet open shell)
CAM-B3LYP /aug-cc-pVTZ= -188.909649979

B	0.000000000000	0.000000000000	-1.531358000000
H	0.000000000000	-1.197201000000	-1.635494000000
H	-1.036807000000	0.598601000000	-1.635494000000
H	1.036807000000	0.598601000000	-1.635494000000
Na	0.000000000000	0.000000000000	1.142115000000

$(NaBH_3)^-$ (triplet)
CAM-B3LYP/aug-cc-pVTZ= -188.901620808

Na	-0.011131000000	-1.067157000000	0.000000000000
B	-0.011131000000	1.461413000000	0.000000000000
H	-0.291171000000	2.623686000000	0.000000000000
H	0.234636000000	0.903985000000	1.043878000000
H	0.234636000000	0.903985000000	-1.043878000000

$(NaBH_3)^-$ (singlet)
wB97x/aug-cc-pVTZ= -188.907359956

B	0.000000000000	0.000000000000	-1.582052000000
H	0.000000000000	1.197497000000	-1.685649000000
H	1.037063000000	-0.598748000000	-1.685649000000
H	-1.037063000000	-0.598749000000	-1.685649000000
Na	0.000000000000	0.000000000000	1.245512000000

$(NaBH_3)^-$ (singlet open shell)
wB97x/aug-cc-pVTZ= -188.913189229

B	0.000000000000	0.000000000000	-1.526542000000

H	0.000000000000	-1.202656000000	-1.642410000000
H	-1.041530000000	0.601328000000	-1.642410000000
H	1.041530000000	0.601328000000	-1.642410000000
Na	0.000000000000	0.000000000000	1.141812000000

$(NaBH_3)^-$ (triplet)
wB97x/aug-cc-pVTZ= -188.906098241

Na	-0.018342000000	-1.066209000000	0.000000000000
B	-0.018342000000	1.448930000000	0.000000000000
H	-0.438834000000	2.574759000000	0.000000000000
H	0.366152000000	0.954447000000	1.045057000000
H	0.366152000000	0.954447000000	-1.045057000000

$(NaBH_3)^-$ (singlet)
B2PLYP/aug-cc-pVTZ= -188.730460491

B	1.579783000000	0.000000000000	0.000004000000
H	1.669236000000	1.014416000000	0.634451000000
H	1.669253000000	-1.056654000000	0.561288000000
H	1.669322000000	0.042242000000	-1.195725000000
Na	-1.173339000000	0.000000000000	-0.000003000000

$(NaBH_3)^-$ (singlet open shell)
B2PLYP/aug-cc-pVTZ=-188.736952122

B	0.000000000000	0.000000000000	-1.641565000000
H	0.000071000000	1.198499000000	-1.726521000000
H	1.037895000000	-0.599311000000	-1.726521000000
H	-1.037966000000	-0.599188000000	-1.726521000000
Na	0.000000000000	0.000000000000	1.090439000000

$(NaBH_3)^-$ (triplet)
B2PLYP/aug-cc-pVTZ=-188.731159229

Na	-0.009098000000	-1.080549000000	0.000000000000
B	-0.009098000000	1.481382000000	0.000000000000
H	-0.304403000000	2.639456000000	0.000000000000
H	0.224987000000	0.919836000000	1.044050000000
H	0.224987000000	0.919836000000	-1.044050000000

$(NaBH_3)^-$ (singlet)
mPW2PYLP/aug-cc-pVTZ=-188.743440023

B	0.000000000000	0.000000000000	-1.583489000000
H	0.000000000000	1.194954000000	-1.671523000000
H	1.034861000000	-0.597477000000	-1.671523000000
H	-1.034861000000	-0.597477000000	-1.671523000000
Na	0.000000000000	0.000000000000	1.175638000000

$(NaBH_3)^-$ (singlet open shell)
mPW2PLYP/aug-cc-pVTZ=-188.749647364

B	0.000000000000	0.000000000000	-1.562607000000
H	0.000000000000	1.197211000000	-1.646403000000
H	1.036815000000	-0.598606000000	-1.646403000000
H	-1.036815000000	-0.598606000000	-1.646403000000
Na	0.000000000000	0.000000000000	1.159295000000

$(NaBH_3)^-$ (triplet)
mPW2PLYP/aug-cc-pVTZ=-188.743497236

Na	-0.009080000000	-1.076760000000	0.000000000000
B	-0.009080000000	1.476519000000	0.000000000000
H	-0.285420000000	2.637998000000	0.000000000000
H	0.215348000000	0.911886000000	1.043210000000
H	0.215348000000	0.911886000000	-1.043210000000

$(NaBH_3)^-$ (singlet)
PBE0DH/aug-cc-pVTZ=-188.736842612

B	0.000000000000	0.000000000000	-1.587670000000
H	0.000000000000	1.198600000000	-1.678945000000
H	1.038018000000	-0.599300000000	-1.678945000000
H	-1.038018000000	-0.599300000000	-1.678945000000
Na	0.000000000000	0.000000000000	1.179562000000

$(NaBH_3)^-$ (singlet open shell)
PBE0DH/aug-cc-pVTZ=-188.745862512

B	0.000000000000	0.000000000000	-1.536423000000
H	0.000000000000	1.202555000000	-1.627599000000
H	1.041443000000	-0.601277000000	-1.627599000000
H	-1.041443000000	-0.601277000000	-1.627599000000
Na	0.000000000000	0.000000000000	1.142265000000

$(NaBH_3)^-$ (triplet)
PBE0DH/aug-cc-pVTZ=-188.741362509

Na	-0.014170000000	-1.069151000000	0.000000000000
B	-0.014170000000	1.459193000000	0.000000000000
H	-0.382378000000	2.599634000000	0.000000000000
H	0.304547000000	0.932528000000	1.045625000000
H	0.304547000000	0.932528000000	-1.045625000000

$(NaBH_3)^-$ (singlet)
PBEQIDH/aug-cc-pVTZ=-188.625955370

B	0.000000000000	0.000000000000	-2.346728000000
H	0.000000000000	1.197683000000	-2.443839000000
H	1.037224000000	-0.598842000000	-2.443839000000
H	-1.037224000000	-0.598842000000	-2.443839000000
Na	0.000000000000	0.000000000000	0.419173000000

$(NaBH_3)^-$ (singlet open shell)
PBEQIDH/aug-cc-pVTZ=-188.638984729

B	0.000000000000	0.000000000000	-1.539076000000
H	0.000000000000	1.201738000000	-1.629689000000
H	1.040736000000	-0.600869000000	-1.629689000000
H	-1.040736000000	-0.600869000000	-1.629689000000
Na	0.000000000000	0.000000000000	1.144041000000

$(NaBH_3)^-$ (triplet)
PBEQIDH/aug-cc-pVTZ=-188.636176988

Na	-0.014341000000	-1.071605000000	0.000000000000
B	-0.014341000000	1.463522000000	0.000000000000
H	-0.346935000000	2.613452000000	0.000000000000
H	0.288198000000	0.928298000000	1.044415000000
H	0.288198000000	0.928298000000	-1.044415000000

BH_4^- (singlet)
CCSD(T)/aug-cc-pVTZ= -27.18622393

B	0.0000000000	0.0000000000	0.0000000000
H	0.7157131235	-0.7157147295	0.7157076109
H	-0.7157131235	-0.7157147295	-0.7157076109
H	0.7157131235	0.7157147295	-0.7157076109
H	-0.7157131235	0.7157147295	0.7157076109

BH_4^- (singlet)
BP86/aug-cc-pVTZ= -27.2676258336

B	0.000000000000	0.000000000000	0.000000000000
H	0.718398000000	0.718398000000	0.718398000000
H	-0.718398000000	-0.718398000000	0.718398000000
H	0.718398000000	-0.718398000000	-0.718398000000
H	-0.718398000000	0.718398000000	-0.718398000000

NH_3BH_3 (singlet)
CCSD(T)/aug-cc-pVTZ= -83.06925736

B	0.0000000000	-0.0000007171	-0.9301897150
H	0.0000000000	-1.1711067261	-1.2384681895
H	-1.0142082651	0.5855531494	-1.2384640811
H	1.0142082651	0.5855531494	-1.2384640811
N	0.0000000000	0.0000007165	0.7271834455
H	0.0000000000	0.9483619775	1.0908516642
H	0.8213048616	-0.4741797749	1.0908519784
H	-0.8213048616	-0.4741797749	1.0908519784

NH_3BH_3 (singlet)
BP86/aug-cc-pVTZ= -83.2452374668

B	0.000000000000	0.000000000000	-0.929419000000
H	0.000000000000	-1.174637000000	-1.245804000000

H	-1.017266000000	0.587319000000	-1.245804000000
H	1.017266000000	0.587319000000	-1.245804000000
N	0.000000000000	0.000000000000	0.727254000000
H	0.000000000000	0.954215000000	1.097910000000
H	0.826374000000	-0.477107000000	1.097910000000
H	-0.826374000000	-0.477107000000	1.097910000000

NH_3 (singlet)
CCSD(T)/aug-cc-pVTZ= -56.48056266

N	0.0000000000	0.0000007210	0.7100049157
H	0.0000000000	0.9383598235	1.0965778407
H	0.8126427463	-0.4691787001	1.0965781550
H	-0.8126427463	-0.4691787001	1.0965781550

NH_3^+ (doublet)
CCSD(T)/aug-cc-pVTZ= -56.10762839

N	0.0000000000	-0.0000120877	0.9999332537
H	0.0000000000	1.0230529043	0.9999391786
H	0.8859667828	-0.5115188362	0.9999333171
H	-0.8859667828	-0.5115188362	0.9999333171

BH_3 (singlet)
CCSD(T)/aug-cc-pVTZ= -26.53908172

B	0.0000000000	-0.0000002394	-1.6722236294
H	0.0000000000	1.1913845751	-1.6722227988
H	1.0317694769	-0.5956926678	-1.6722227859
H	-1.0317694769	-0.5956926678	-1.6722227859

BH_3 (Doublet)
CCSD(T)/aug-cc-pVTZ= -26.53606725

B	0.0064960229	0.0093291630	-1.5777719917
H	-0.0200886138	1.2139548533	-1.4574325077
H	1.0663182997	-0.5764357775	-1.5748579428
H	-1.0272057470	-0.6092393683	-1.7014255753

References

[1] (a) A. D. Becke, *Phys. Rev. A* **1988**, *38*, 3098-3100; (b) J. P. Perdew, *Phys. Rev. B* **1986**, *33*, 8822-8824.

[2] C. Adamo, V. Barone, *J. Chem. Phys.* **1999**, *110*, 6158-6170.

[3] (a) A. D. Becke, *J. Chem. Phys.* **1993**, *98*, 5648-5652; (b) C. Lee, W. Yang, R. G. Parr, *Phys. Rev. B* **1988**, *37*, 785-789.

[4] J. M. Tao, J. P. Perdew, V. N. Staroverov, G. E. Scuseria, *Phys. Rev. Lett.* **2003**, *91*, 146401-146404.

[5] Y. Zhao, D. G. Truhlar, *J. Chem. Phys.* **2006**, *125*.

[6] X. Xu, W. A. Goddard, *Proc. Natl. Acad. Sci. U.S.A.* **2004**, *101*, 2673-2677.

[7] M. Ernzerhof, G. E. Scuseria, *J. Chem. Phys.* **1999**, *110*, 5029-5036.

[8] C. Adamo, V. Barone, *J. Chem. Phys.* **1998**, *108*, 664-675.

[9] A. D. Becke, *J. Chem. Phys.* **1993**, *98*, 1372-1377.

[10] (a) V. N. Staroverov, G. E. Scuseria, J. M. Tao, J. P. Perdew, *J. Chem. Phys.* **2003**, *119*, 12129-12137; (b) V. N. Staroverov, G. E. Scuseria, J. M. Tao, J. P. Perdew, *J. Chem. Phys.* **2004**, *121*, 11507-11507.

[11] Y. Zhao, D. G. Truhlar, *Theo. Chem. Acc.* **2008**, *120*, 215-241.

[12] T. Yanai, D. P. Tew, N. C. Handy, *Chem. Phys. Lett.* **2004**, *393*, 51-57.

[13] J.-D. Chai, M. Head-Gordon, *J. Chem. Phys.* **2008**, *128*, 084106.

[14] S. Grimme, *J. Chem. Phys.* **2006**, *124*.

[15] T. Schwabe, S. Grimme, *Phys. Chem. Chem. Phys.* **2006**, *8*, 4398-4401.

[16] E. Brémond, C. Adamo, *J. Chem. Phys.* **2011**, *135*, 024106.

[17] É. Brémond, J. C. Sancho-García, Á. J. Pérez-Jiménez, C. Adamo, *J. Chem. Phys.* **2014**, *141*, 031101.

[18] (a) T. H. Dunning Jr, *J. Chem. Phys.* **1989**, *90*, 1007-1023; (b) R. A. Kendall, T. H. Dunning, R. J. Harrison, *J. Chem. Phys.* **1992**, *96*, 6796-6806; (c) D. E. Woon, T. H. Dunning Jr, *J. Chem. Phys.* **1993**, *98*, 1358-1371; (d) D. E. Woon, T. H. Dunning Jr, *J. Chem. Phys.* **1994**, *100*, 2975-2988.

[19] M. J. Frisch, G. W. Trucks, H. B. Schlegel, G. E. Scuseria, M. A. Robb, J. R. Cheeseman, G. Scalmani, V. Barone, G. A. Petersson, H. Nakatsuji, X. Li, M. Caricato, A. V. Marenich, J. Bloino, B. G. Janesko, R. Gomperts, B. Mennucci, H. P. Hratchian, J. V. Ortiz, A. F. Izmaylov, J. L. Sonnenberg, D. Williams-Young, F. Ding, F. Lipparini, F. Egidi, J. Goings, B. Peng, A. Petrone, T. Henderson, D. Ranasinghe, V. G. Zakrzewski, J. Gao, N. Rega, G. Zheng, W. Liang, M. Hada, M. Ehara, K. Toyota, R. Fukuda, J. Hasegawa, M. Ishida, T. Nakajima, Y. Honda, O. Kitao, H. Nakai, T. Vreven, K. Throssell, J. J. A. Montgomery, J. E. Peralta, F. Ogliaro, M. J. Bearpark, J. J. Heyd, E. N. Brothers, K. N. Kudin, V. N. Staroverov, T. A. Keith, R. Kobayashi, J. Normand, K. Raghavachari, A. P. Rendell, J. C. Burant, S. S. Iyengar, J. Tomasi, M. Cossi, J. M. Millam, M. Klene, C. Adamo, R. Cammi, J. W. Ochterski, R. L. Martin, K. Morokuma, O. Farkas, J. B. Foresman, D. J. Fox, Gaussian 16, Revision C.01. Gaussian, Inc., Wallingford CT, **2019**.

[20] C. Y. Peng, P. Y. Ayala, H. B. Schlegel, M. J. Frisch, *J. Comp. Chem.* **1996**, *17*, 49-56.

[21] J. W. McIver, Komornic.A, *J. Am. Chem. Soc.* **1972**, *94*, 2625-2633.

[22] P. W. Atkins, J. De Paula, *Physical Chemistry. 8th ed.*, Oxford: Oxford University Press, New York, **2006**.

[23] H.-J. Werner, P. J. Knowles, G. Knizia, F. R. Manby, M. Schütz, P. Celani, T. Korona, R. Lindh, A. Mitrushenkov, G. Rauhut, K. R. Shamasundar, T. B. Adler, R. D. Amos, A. Bernhardsson, A. Berning, D. L. Cooper, M. J. O. Deegan, A. J. Dobbyn, F. Eckert, E. Goll, C. Hampel, A. Hesselmann, G. Hetzer, T. Hrenar, G. Jansen, C. Köppl, Y. Liu, A. W. Lloyd, R. A. Mata, A. J. May, S. J. McNicholas, W. Meyer, M. E. Mura, A. Nicklass, D. P. O'Neill, P. Palmieri, D. Peng,

K. Pflüger, R. Pitzer, M. Reiher, T. Shiozaki, H. Stoll, A. J. Stone, R. Tarroni, T. Thorsteinsson, M. Wang, http://www.molpro.net., **2012**.
[24] T. Shiozaki, *WIREs Computational Molecular Science* **2018**, *8*, e1331.
[25] O. Tishchenko, J. Zheng, D. G. Truhlar, *J. Chem. Theory Comput.* **2008**, *4*, 1208-1219.
[26] Q. Sun, T. C. Berkelbach, N. S. Blunt, G. H. Booth, S. Guo, Z. Li, J. Liu, J. D. McClain, E. R. Sayfutyarova, S. Sharma, S. Wouters, G. K.-L. Chan, *WIREs Computational Molecular Science* **2018**, *8*, e1340.
[27] P. Salvador, E. Ramos-Cordoba, Universitat de Girona, Girona, Spain, **2012**.
[28] G. te Velde, F. M. Bickelhaupt, E. J. Baerends, C. Fonseca Guerra, S. J. A. van Gisbergen, J. G. Snijders, T. Ziegler, *J. Comp. Chem.* **2001**, *22*, 931-967.
[29] J. Krijn, E. J. Baerends, *Fit Functions in the HFS-Method* **1984**.
[30] E. Van Lenthe, E. J. Baerends, J. G. Snijders, *J. Chem. Phys.* **1993**, *99*, 4597-4610.
[31] V. Bachler, G. Olbrich, F. Neese, K. Wieghardt, *Inorg. Chem.* **2002**, *41*, 4179-4193.
